# Supplementary material for: COVID-19 vaccines uptake: Public knowledge, awareness, perception and acceptance among adult Africans
Source: PLoS One. 2022 Jun 1;17(6):e0268230. doi: 10.1371/journal.pone.0268230 (PMC9159554; doi:10.1371/journal.pone.0268230)
Supplement: Attachment — Submitted filename: my Comments.docx [file pone.0268230.s002.docx]

**Manuscript ID- PONE-D-21-36946**

The manuscript is well written, and it can be accepted after the minor revision. Please find my comments below.

1. Various symptoms and prevention strategy of Covid-19 should be provided.

2. The variant of Covid-19 are measure concern for the treatment of infected patients. so, few more information may be provided.

3. Please provide include the data how the various vaccine is effective for the treatment of Covid-19 and their variant in the discussion section.

4. please illustrate or highlighted the summary for the significant of present study (Add 1or 2 figures).

5. Please combine 1-3 figures in one figure as they are very small.
