# Supplementary material for: COVID-19 vaccines uptake: Public knowledge, awareness, perception and acceptance among adult Africans
Source: PLoS One. 2022 Jun 1;17(6):e0268230. doi: 10.1371/journal.pone.0268230 (PMC9159554; doi:10.1371/journal.pone.0268230)
Supplement: Attachment — Submitted filename: Authors response to review comments.docx [file pone.0268230.s003.docx]

PONE-D-21-36946

COVID-19 vaccines uptake: Public knowledge, awareness, perception and acceptance among adult Africans

PLOS ONE

Dear Dr. Ahiakpa,

Thank you for submitting your manuscript to PLOS ONE. After careful consideration, we feel that it has merit but does not fully meet PLOS ONE’s publication criteria as it currently stands. Therefore, we invite you to submit a revised version of the manuscript that addresses the points raised during the review process.

Please submit your revised manuscript by Feb 19 2022 11:59PM. If you will need more time than this to complete your revisions, please reply to this message or contact the journal office at plosone@plos.org. When you're ready to submit your revision, log on to https://www.editorialmanager.com/pone/ and select the 'Submissions Needing Revision' folder to locate your manuscript file.

Please include the following items when submitting your revised manuscript:

•	A rebuttal letter that responds to each point raised by the academic editor and reviewer(s). You should upload this letter as a separate file labeled 'Response to Reviewers'.

•	A marked-up copy of your manuscript that highlights changes made to the original version. You should upload this as a separate file labeled 'Revised Manuscript with Track Changes'.

•	An unmarked version of your revised paper without tracked changes. You should upload this as a separate file labeled 'Manuscript'.

If you would like to make changes to your financial disclosure, please include your updated statement in your cover letter. Guidelines for resubmitting your figure files are available below the reviewer comments at the end of this letter.

If applicable, we recommend that you deposit your laboratory protocols in protocols.io to enhance the reproducibility of your results. Protocols.io assigns your protocol its own identifier (DOI) so that it can be cited independently in the future. For instructions see: https://journals.plos.org/plosone/s/submission-guidelines#loc-laboratory-protocols. Additionally, PLOS ONE offers an option for publishing peer-reviewed Lab Protocol articles, which describe protocols hosted on protocols.io. Read more information on sharing protocols at https://plos.org/protocols?utm_medium=editorial-email&utm_source=authorletters&utm_campaign=protocols.

We look forward to receiving your revised manuscript.

Kind regards,

Sanjay Kumar Singh Patel, Ph.D.

Academic Editor

PLOS ONE

Dear Editor,

We are grateful to you, and the rreviewers for giving us the opportunity to improve the scientific quality of our manuscript. We have revised the manuscript and prepared point-by-point responses to the reviewers’ comments. We look forward to your decision soon.

Thank you.

Journal Requirements:

When submitting your revision, we need you to address these additional requirements.

1. Please ensure that your manuscript meets PLOS ONE's style requirements, including those for file naming. The PLOS ONE style templates can be found at

https://journals.plos.org/plosone/s/file?id=wjVg/PLOSOne_formatting_sample_main_body.pdf and

<https://journals.plos.org/plosone/s/file?id=ba62/PLOSOne_formatting_sample_title_authors_affiliations.pdf>.

**#Authors’ response:** We revised the sections are recommended.

2. During our internal checks, the in-house editorial staff noted that you conducted research or obtained samples in another country. Please check the relevant national regulations and laws applying to foreign researchers and state whether you obtained the required permits and approvals. Please address this in your ethics statement in both the manuscript and submission information. In addition, please ensure that you have suitably acknowledged the contributions of any local collaborators involved in this work in your authorship list and/or Acknowledgements. Authorship criteria is based on the International Committee of Medical Journal Editors (ICMJE) Uniform Requirements for Manuscripts Submitted to Biomedical Journals - for further information please see here: <https://journals.plos.org/plosone/s/authorship>.

**#Authors’ response:** The study was conducted online using the chain-referral approach where participants voluntarily participated in the survey. Consortium members/co-authors in the respective countries didn’t have any influence in the participation of the study. Thus, the voluntary nature and non-geographical specificity of the study preclude from obtaining ethical clearance from each country. Thus, a recognised ethical clearance from the study’s origin, Somalia should be adequate.

3. Thank you for stating the following financial disclosure:

[We had no funding for this study].

At this time, please address the following queries:

a) Please clarify the sources of funding (financial or material support) for your study. List the grants or organizations that supported your study, including funding received from your institution.

**#Authors’ response:** We didn’t receive any funding from any agency.

b) State what role the funders took in the study. If the funders had no role in your study, please state: “The funders had no role in study design, data collection and analysis, decision to publish, or preparation of the manuscript.”

**#Authors’ response:** There is was no funding for this study

c) If any authors received a salary from any of your funders, please state which authors and which funders.

**#Authors’ response:** This is not applicable

d) If you did not receive any funding for this study, please state: “The authors received no specific funding for this work.”

Please include your amended statements within your cover letter; we will change the online submission form on your behalf.

**#Authors’ response:** The authors received no specific funding for this work

4. We note you have included a table to which you do not refer in the text of your manuscript. Please ensure that you refer to Table 4 in your text; if accepted, production will need this reference to link the reader to the Table.

**#Authors’ response:** We have cited the table in the revised manuscript. See L268-L300 in the revised manuscript.

5. Please review your reference list to ensure that it is complete and correct. If you have cited papers that have been retracted, please include the rationale for doing so in the manuscript text, or remove these references and replace them with relevant current references. Any changes to the reference list should be mentioned in the rebuttal letter that accompanies your revised manuscript. If you need to cite a retracted article, indicate the article’s retracted status in the References list and also include a citation and full reference for the retraction notice.

**#Authors’ response:** We have reviewed the reference list and updated it where necessary

**Response to Review Comments**

Dear Editor,

We are submitting the revised version of our manuscript with point-by-point responses to the reviewers’ comments. We are grateful to you, and the reviewers for the opportunity to improve the scientific quality/rigour of our manuscript. Below are point-by-point responses to issues raised by the reviewers and yourself. Corrections were directly effected in the manuscript in track changes.

Thank you.

Reviewers' comments:

Reviewer's Responses to Questions

Comments to the Author

1. Is the manuscript technically sound, and do the data support the conclusions?

The manuscript must describe a technically sound piece of scientific research with data that supports the conclusions. Experiments must have been conducted rigorously, with appropriate controls, replication, and sample sizes. The conclusions must be drawn appropriately based on the data presented.

Reviewer #1: Yes

Reviewer #2: Yes

**#Authors’ response:** Thank you for your assessment and comments

2. Has the statistical analysis been performed appropriately and rigorously?

Reviewer #1: Yes

Reviewer #2: Yes

**#Authors’ response:** Thank you for your comments

3. Have the authors made all data underlying the findings in their manuscript fully available?

The PLOS Data policy requires authors to make all data underlying the findings described in their manuscript fully available without restriction, with rare exception (please refer to the Data Availability Statement in the manuscript PDF file). The data should be provided as part of the manuscript or its supporting information, or deposited to a public repository. For example, in addition to summary statistics, the data points behind means, medians and variance measures should be available. If there are restrictions on publicly sharing data—e.g. participant privacy or use of data from a third party—those must be specified.

Reviewer #1: Yes

Reviewer #2: Yes

**#Authors’ response:** Thank you for the comments

4. Is the manuscript presented in an intelligible fashion and written in standard English?

PLOS ONE does not copyedit accepted manuscripts, so the language in submitted articles must be clear, correct, and unambiguous. Any typographical or grammatical errors should be corrected at revision, so please note any specific errors here.

Reviewer #1: Yes

Reviewer #2: Yes

**#Authors’ response:** We appreciate the comments

5. Review Comments to the Author

Please use the space provided to explain your answers to the questions above. You may also include additional comments for the author, including concerns about dual publication, research ethics, or publication ethics. (Please upload your review as an attachment if it exceeds 20,000 characters)

Reviewer #1: In this paper entitled "COVID-19 vaccines uptake: Public knowledge, awareness, perception and acceptance among adult Africans", the authors investigated adult African inhabitant's views toward vaccination and their participation possibility in governments efforts to get citizens vaccinated. Adult Africans' responses were taken in the manuscript using a cross-sectional online survey, and the factors associated with willingness to accept the COVID-19 vaccine and participation were evaluated. The results indicated that more than two-thirds of African adults would not receive the COVID-19 vaccine as they have skepticisms about it. The manuscript is easy to understand and technically correct. Furthermore, the manuscript is statistically sound and has potential. Therefore, it may be considered for publication after minor corrections.

**#Authors’ response:** We appreciate the comments and recommendation

Minor comments:

1) The English may be improved for the manuscript.

**#Authors’ response:** We have revised the manuscript where necessary.

2) In the title, define adult Africans?. The authors should cross-check all abbreviations in the manuscript. Initially, define in the full name followed by abbreviations.

**#Authors’ response:** We defined the category of participants in the study in the methodology section. See L139 in the revised manuscript.

3) Introduction section may be minor polished with information such as - i) COVID-19 details, symptoms and prevention strategies including health status and diet i.e. doi: 10.1007/s12088-020-00908-0; doi: 10.1007/s12088-020-00893-4; ii) about COVID-19 variants and their future challenges i.e. doi: 10.1007/s15010-021-01734-2.

**#Authors’ response:** We defined the category of participants in the study in the methodology section. See L139 in the revised manuscript. We have equally updated the introduction with the specific recommendations. See L79-94; L100-L107; and L126-144 in the revised manuscript.

4) Design of the study and participants: How the author determines the number of participants? Which reference study or formula is used to calculate the participant number for the study?

**#Authors’ response:** The study was conducted using random selection process with a cross-sectional sampling (snowballing) or chain-referral approach where participants voluntarily participated in the survey. The conventional Cochran formula [29] was used to determine the starting sample size;

no = $\frac{Z2\mathrm{pq}}{e2}$,

where e = the desired precision level (margin of error), where p is the fraction of population, q is 1-p, and Z is the Z-value found in a Z table. A total of 365 participants completed the closed-ended questionnaire for our study. At a 95% level of confidence, this corresponds to a 2 % margin of error [29]. See L210-227

5) Introduction, the importance of this study may be more specifically highlighted.

**#Authors’ response:** We stated the relevance of the study specifically in the introduction. See lines 111-129.

7) The author may provide a paragraph regarding challenges or prospects of study in the discussion and provide a limitation of the study.

**#Authors’ response:** We have rephrased this in the discussion section as recommended. See L495-501

Reviewer #2:

1. Various symptoms and prevention strategy of Covid-19 should be provided.

**#Authors’ response:** We have incorporated this in the revised manuscript

2. The variant of Covid-19 are measure concern for the treatment of infected patients. so, few more information may be provide.

**#Authors’ response:** We have included information on this in the revised manuscript

3. Please provide include the data how the various vaccine is effective for the treatment of Covid-19 and their variant in the discussion section.

**#Authors’ response:** We provided percentage efficacies of the various vaccines in the introduction. See lines 126-137.

4. please illustrate or highlighted the summary for the significant of present study (Add 1or 2 figures).

**#Authors’ response:** We have incorporated this in the revised manuscript. See L495-501.

5. Please combine 1-3 figures in one figure as they are very small.

**#Authors’ response:** We have merged these figures as recommended. Thank you.
